# Abnormal Glucose Metabolism Parameters and the Aggressiveness of Differentiated Thyroid Carcinoma: A Hospital-Based Cross-Section Study in China

**DOI:** 10.3389/fendo.2022.806349

**Published:** 2022-03-01

**Authors:** Junyu Zhao, Yutian Tian, Zhen Jia, Jinming Yao, Lin Liao, Jianjun Dong

**Affiliations:** ^1^ Department of Endocrinology and Metabology, The First Affiliated Hospital of Shandong First Medical University & Shandong Provincial Qianfoshan Hospital, Shandong Key Laboratory of Rheumatic Disease and Translational medicine, Shandong Institute of Nephrology, Jinan, China; ^2^ Department of Endocrinology and Metabology, Shandong Provincial Qianfoshan Hospital, Cheeloo College of Medicine, Shandong University, Jinan, China; ^3^ Division of Endocrinology, Department of Endocrinology, Qilu Hospital, Cheeloo College of Medicine, Shandong University, Jinan, China

**Keywords:** differentiated thyroid carcinoma, area under curve, insulin sensitivity index, insulin resistance, homeostasis model assessment of insulin resistance

## Abstract

**Purpose:**

The correlation of abnormal glucose metabolism and thyroid carcinoma, especially the aggressiveness of thyroid cancer, still remains controversial. We conducted this study to investigate the relationship between abnormal glucose metabolism parameters and differentiated thyroid carcinoma (DTC) in the Chinese population.

**Materials and Methods:**

The study was designed as a hospital-based case–control study and was approved by the Ethics Committee of our hospital and registered in the Clinical Trial Protocol Registration and Results System (Registration code: NCT 03006289). From January 1, 2018 to June 30, 2021, a total of 377 DTC patients were enrolled in the study. Demographic and general characteristics, details of thyroid surgery and histopathological results, hematological test indicators were collected. Glucose metabolism parameters were calculated. Variables were analyzed by t-test, ANOVA, chi-squared analysis and Fisher’s exact test. Pearson bi-variate correlation and Spearman’s correlation analysis were used for bi-variate analysis.

**Results:**

More than 40% of patients with DTC were multifocality, more than half were extra-glandular invasion, and nearly 85% complied by lymph node metastasis. The prevalence of diabetes mellitus (DM) was about 10.08% in DTC patients. It was found that the proportion of postprandial 2 h blood glucose ≥11.1mmol/L and HbA1c ≥6.5% was significantly higher than the known proportion of DM (17.8%, 16.7% vs. 10.08%). Additionally, 87.3% of the DTC patients in this study had varying degrees of insulin resistance. Further analysis found that higher T staging was associated with higher levels of area under curve of C-peptide (*P* = 0.029), insulin sensitivity index (*P* = 0.012) and C-peptide sensitivity index (*P* = 0.016). A delayed peak of insulin secretion was found to be positive related with capsule invasion (r = 0.206, *P* = 0.004). In patients without a DM history, homeostasis model assessment of insulin resistance (*P* = 0.017), insulin sensitivity index (*P* = 0.019) and C-peptide sensitivity index (*P* = 0.020) were statistic associated with T staging. Also, the glucose metabolism parameter at 3-hour after a meal was related to a larger number of metastatic lymph nodes.

**Conclusion:**

Abnormal glucose metabolism, namely, DM, hyperinsulinemia and insulin resistance, were significantly associated with the carcinogensis and aggressiveness of DTC.

## Introduction

Thyroid carcinoma is the most common tumor of the endocrinology systematic diseases, with an annual increasing incidence, making it one of the fastest growing cancers worldwide (1). It increased with an average annual growth rate of 12.4% (95% CI: 10.5–14.4%) in China from 2005 to 2015 (2). Our previous study showed that papillary thyroid carcinoma (PTC) accounted for 98.4% of all thyroid malignant tumors in Shandong, China, among which 39.21% patients were multiple lesions and 46.86% patients were accompanied by metastatic lymph node(s) (3). Although the majority of the patients have a good prognosis after a systematic treatment and follow-up, nearly 15–20% of the patients are still likely to experience recurrence despite an active treatment, and the 10-year survival rate will be significantly reduced once recurrence occurs (4). Meanwhile, the characteristics of multiple lesions and early lymph node metastasis in PTC patients bring a heavy psychological and economic burden to patients, families and the society. Therefore, controlling the factors that could have impacted the invasion and metastasis or even the occurrence of thyroid carcinoma, could improve the prognosis of the diseases. So, an investigation of its risk factor is of great significance for the prevention and treatment of thyroid carcinoma.

Except for the history of radiation exposure, most of the currently known risk factors for tumorigenesis and poorly prognosis are ineluctable, namely, advanced age, gender, race and family history of thyroid carcinoma. Some scholars have found that there are other risk factors for thyroid carcinoma. Dal Maso et al. (5) showed a positive association between body-mass index (BMI) and thyroid carcinoma risk. NCD Risk Factor Collaboration and several studies reported the relationship between obesity and thyroid carcinoma risk (6, 7), and even increased tumor growth and promoted anaplastic change in a mouse model (8). Studies on glucose metabolism showed that both prediabetes (impaired fasting glucose and impaired glucose tolerance) and diabetes were strongly associated with thyroid malignancy and poor prognosis (9, 10). Recently, some scientists proposed that hyperinsulinemia or insulin resistance might be associated with thyroid carcinoma (11–13), while others have not found the relationship (14). Whether hyperinsulinemia or insulin resistance is related to thyroid carcinoma is still inconsistent. In addition, most of the current study focuses on the risk of thyroid cancer, and the aggressiveness of diseases was not considered. So, we conducted a cross-sectional study to investigate the associations between the glucose metabolism and its related parameters and the aggressiveness of thyroid carcinoma.

## Materials and Methods

### Study Design and Objects

This cross-sectional study included patients with differentiated thyroid carcinoma (DTC) who were scheduled to receive iodine-131 treatment in the First Affiliated Hospital of Shandong First Medical University (Shandong Provincial Qianfoshan Hospital) from January 1, 2018 to June 30, 2021. The patients had completed surgical pathological details and the glucose metabolism indicators were tested after thyroidectomy surgery but before iodine-131 treatment during the hospitalization to receive the iodine-131 treatment. The inclusion and exclusion criteria of this study are as follows:

Inclusion criteria:

(1) Study time: From January 1, 2018 to June 30, 2021;
(2) Study site: The First Affiliated Hospital of Shandong First Medical University (Shandong Provincial Qianfoshan Hospital);
(3) Surgical methods: Total thyroidectomy with or without lymph node dissection;
(4) Histopathological results: clearly diagnosed as DTC or lymph node metastasis from thyroid follicular cells, all with completed pathological details;
(5) Completed the detection of glucose metabolism indicators (namely, oral glucose tolerance test and insulin/C-peptide release test).

Exclusion criteria:

(1) The pathological type was not DTC;
(2) Lack of surgical pathological details;
(3) Other tumors metastasized to the thyroid;
(4) Non-Chinese patients;
(5) Patients during pregnancy or lactation;
(6) Incomplete clinical data.

There were no differences based on gender or race/ethnicity and the trial was conducted in accordance with guidelines and protocols associated with clinical studies. This study protocol was approved by the Ethics Committee of our hospital and registered in the Clinical Trial Protocol Registration and Results System (Registration code: NCT 03006289).

### Data Collection

We collected and recorded each demographic and general characteristics of patients, namely, gender, age, height, weight, smoking and drinking history, history of chronic diseases (DM, hypertension, hyperlipidemia, hyperthyroidism, hypothyroidism, Hashimoto’s thyroiditis), details of thyroid surgery (total thyroidectomy with or without lymph node dissection) and histopathological results (type of pathology, tumor size, number of metastatic lymph nodes, whether the lesion was unilateral or bilateral. whether the lesion was multifocality, whether the lesion capsule invasion, and if there a metastasis), and TNM (tumor, node, metastasis) staging performed according to pathological details (American Joint Committee on Cancer, AJCC 8th edition). Clinical hematological test indicators: thyroglobulin (Tg), antithyroglobulin antibody (Tg-Ab), thyroid stimulating hormone (TSH), glycosylated hemoglobin (HbA1c), triglycerides (TG), total cholesterol (TC), low-density lipoprotein cholesterol (LDL-c), high-density lipoprotein cholesterol (HDL-c), uric acid (UA), plasma glucose (fasting, 1 h, 2 h, 3 h), level of insulin (fasting, 1 h, 2 h, 3 h), and level of C-peptide (fasting, 1 h, 2 h, 3 h). All the information above can be queried in the electronic medical record system of The First Affiliated Hospital of Shandong First Medical University (Shandong Provincial Qianfoshan Hospital). Systolic blood pressure (SBP) and diastolic blood pressure (DBP) were also measured at the time when collecting pathological data and clinical data. BMI, homeostasis model assessment of β cell (HOMA-β), homeostasis model assessment of insulin resistance (HOMA-IR), insulin sensitivity index (ISI), and C-peptide sensitivity index (CSI) were calculated as well and the formulas are showed below:

BMI (kg/m^2^) = weight (kg) / height^2^ (m).
HOMA-β = Fasting Serum Insulin (μIU/ml) × 20 / (Fasting Plasma Glucose (mmol/L − 3.5).
HOMA-IR = Fasting Serum Insulin (μIU/ml) × Fasting Plasma Glucose (mmol/l) / 22.5.
ISI = 10,000/(Fasting Plasma Glucose (mmol/l) × 10)^1/2^(Mean Plasma Glucose (mmol/l) × Mean Serum Insulin (μIU/ml))^1/2^.
CSI = 10,000/(Fasting Plasma Glucose (mmol/l) × 10)^1/2^ (Mean Plasma Glucose (mmol/l) × Mean Serum C-peptide (μIU/ml))^1/2^.
AUC of insulin = Fasting Serum Insulin/2 + 1 h Serum Insulin + 2 h Serum Insulin + 3 h Serum Insulin/2 (μIU/ml).
AUC of C-peptide = Fasting Serum C-peptide/2 + 1 h Serum C-peptide + 2 h Serum C-peptide + 3 h Serum C-peptide/2 (μIU/ml).

### Statistical Analysis

All statistical analyses were performed using the SPSS 22.0 software (IBM’s Statistical Product and Service Solutions). Measurement data were expressed by mean ± standard deviation, and count data were expressed by frequency and percentage. Variables were analyzed by t-test, ANOVA, chi-squared analysis and Fisher’s exact test. Pearson bi-variate correlation and Spearman’s correlation analysis were used for bi-variate analysis, representing statistical differences as *P <*0.05. The former was used to analyze the correlation between pathological features and glucose metabolism and related parameters, and Spearman’s correlation analysis was performed after statistical differences were found. Meanwhile, data for Spearman’s correlation analysis were adjusted for sex, age, body weight, height, BMI, SBP, DBP, history of DM, duration of DM, smoking, drinking, HbA1c, TG, TC, LDL-c, HDL-c, uric acid, TSH, Tg, and TgAb.

## Results

### General Characteristics

A total of 377 DTC patients (mean age: 44.8 ± 11.9 years), scheduled to receive radioactive iodine-131 treatment, were enrolled in this cross-section study. Demographic and clinical data of these patients are shown in **Table 1**. Of these patients, 36.1% were men and 63.9% were women. The mean BMI of the 377 patients was 26.8 ± 4.3 (range from 15.9 to 43.7). Among them, 145 (38.5%) patients were overweight and 130 (34.5%) patients were obese. Seventy-four (19.6%) patients had known hypertension. The mean SBP and DBP were 128.0 ± 15.4 (range from 86 to 177) and 81.2 ± 11.4 (range from 53 to 113) separately. The total number of patients with abnormal blood pressure was 118 (31.3%), and 87 patients with SBP exceeding than140 mmHg and 80 patients with DBP higher than 90 mmHg. In addition, thirty-eight (10.1%) patients had known DM.

**Table 1:** Demographic and clinical data observed in the cross-section study.

Clinical factors	Data
Gender (N, %)	
Male	136 (36.1)
Female	241 (63.9)
Age (Years), mean ± SD	44.8 ± 11.9
Height (cm), mean ± SD	165.4 ± 7.9
Male	172.7 ± 6.2
Female	161.3 ± 5.5
Weight (kg), mean ± SD	73.8 ± 15.6
Male	83.8 ± 15.7
Female	68.2 ± 12.4
BMI (kg/m^2^), mean ± SD	26.8 ± 4.3
Underweight, < 18.0 (N, %)	1 (0.3)
Normal weight, 18.0 ≤ BMI < 24.0 (N, %)	101 (26.8)
Overweight, 24.0 ≤ BMI < 28.0 (N, %)	145 (38.5)
Obese, ≥ 28.0 (N, %)	130 (34.5)
SBP (mmHg), mean ± SD	128.0 ± 15.4
≥ 140 mmHg (N, %)	87 (23.1)
DBP (mmHg), mean ± SD	81.2 ± 11.4
≥ 90 mmHg (N, %)	80 (21.2)
Medical history	
DM (N, %)	38 (10.1)
Hypertension (N, %)	74 (19.6)
Hperlipidemia (N, %)	6 (1.6)
Smoking (N, %)	12 (3.2)
Male (N, %)	11 (2.9)
Female (N, %)	1 (0.3)
Drinking (N, %)	8 (2.1)
Male (N, %)	7 (1.8)
Female (N, %)	1 (0.3)

SD, standard deviation; BMI, body-mass index; SBP, systolic blood pressure; DBP, diastolic blood pressure; DM, diabetes mellitus.

### Pathological Features of These Patients With DTC

Among the 377 DTC patients, 372 (98.6%) patients were diagnosed as PTC and 4 (1.1%) patients as FTC, the diagnosis of the remaining one was not mentioned in the original article but a Tg positive staining was found on the surface of tumor cells which indicated that the tumor cells were well differentiated. One hundred and fifty-eight (41.9%) patients were bilateral and 166 (44.0%) patients were multifocality. More than half (50.7%) of the patients were found to have extrathyroidal extension. The tumor size was defined by the maximum diameter of the primary lesion and the mean tumor size (cm) was 1.4 ± 1.0 (range from 0.05 to 8.50). Approximately 84.6% patients had lymph node metastasis and the mean number of metastatic lymph nodes was 7.0 ± 6.8 (range from 1 to 43). Most of patients in this study had lymph node metastasis, while metastasis to other tissues and organs, namely, lung and bone metastasis, was rare. Therefore, TNM staging was performed on the included population according to the staging criteria of the AJCC 8th edition, and both T and N staging were finally analyzed without M staging. T staging results showed that nearly 80% of patients had stages I and II. At the same time, almost all of the patients with lymph node metastasis included in the study were stage I by N staging. The details of the pathological features are listed in **Table 2**.

**Table 2:** Pathological features of enrolled patients.

Characteristics of pathology	Results
Pathological types of thyroid neoplasms	
PTC	372 (98.6%)
FTC	4 (1.1%)
Not mentioned	1 (0.3%)
Bilateral	
Unilateral lesion	208 (55.2%)
Bilateral lesion	158 (41.9%)
Not mentioned	11 (2.9%)
Multifocality	
Yes	166 (44.0%)
No	206 (54.7%)
Without primary lesion in thyroid	5 (1.3%)
Capsule invasion	
Yes	191 (50.7%)
No	122 (32.4%)
NA	64 (16.9%)
Tumor size (cm), mean ± SD (range)	1.4 ± 1.0
Metastasis	
Yes	319 (84.6%)
No	56 (14.9%)
Not mentioned	2 (0.5%)
Number of metastatic lymph nodes, mean ± SD (range)	7.0 ± 6.8
TNM staging (AJCC 8th edition)	
T	
Ia	142 (37.7%)
Ib	118 (31.3%)
II	40 (10.6%)
IIIa	3 (0.8%)
IIIb	18 (4.8%)
Iva	15 (4.0%)
IVb	2 (0.5%)
NA	39 (10.3%)
N	
0	56 (14.9%)
Ia	164 (43.5%)
Ib	155 (41.1%)
NA	2 (0.5%)

PTC, thyroid papillary carcinoma; FTC, thyroid follicular carcinoma; TNM, tumor, node, metastasis; AJCC, American Joint Committee on Cancer; SD, standard deviation.

### Glucose Metabolism and Related Parameters

All the enrolled DTC patients received oral glucose tolerance test (OGTT) of 75 g glucose. **Table 3** illustrates their glucose metabolism and related parameters. Patients enrolled were all scheduled to receive radioactive iodine-131 treatment, so the level of TSH was required to be above or near 30 uIU/ml (mean ± SD: 68.0 ± 24.6; range: 24.93–150). The mean HbA1c was 6.0%, and the HbA1c levels of 63 patients (16.7%) were ≥6.5%. This was significantly different from the proportion with known DM (16.7% vs. 10.1%). Among these, 38 (10.1%) patients had no history of DM but their HbA1c exceeded than 6.5%, indicating that 10.1% of diabetes were found at this OGTT. Results of insulin/C-peptide release test were shown below. Out of 377 patients, 17 patients (4.5%) had fasting blood glucose greater than 7.0 mmol/L, whereas, 67 (17.8%) patients had 2 h postprandial blood glucose above 11.1mmol/L. In general, fasting plasma insulin levels should not be higher than 15 uIU/ml, and fasting hyperinsulinemia in people with normal blood glucose is considered a marker of insulin resistance. In the study, there were 64 people (17.0%) whose fasting insulin levels were greater than 15 uIU/ml. An HOMA-IR between 1 and 2.5 was considered as early insulin resistance, and higher than 2.5 was considered as significant insulin resistance. In this cross-section study, 87.3% of the patients had varying degrees of insulin resistance (50.7%–early insulin resistance, 36.6%—significant insulin resistance). In addition, the peak of insulin secretion was delayed in 235 (62.3%) patients, and 280 (74.3%) patients were found with a peak delay of C-peptide secretion.

**Table 3:** Glucose metabolism and related parameters of enrolled patients.

Parameters	Results	
	Mean ± SD	Range
Tg (ng/ml)	27.2 ± 88.0	0.04–500
TgAb (IU/ml)	143.1 ± 533.3	3.91–4,000
TSH (uIU/ml)	68.0 ± 24.6	24.93–150
TG (mmol/L)	2.2 ± 1.9	0.5–21.47
TC (mmol/L)	5.8 ± 1.4	1.16–13.03
LDL-c (mmol/L)	2.6 ± 1.2	0.72–5.86
HDL-c (mmol/L)	2.1 ± 1.2	0.79–6.1
Uric acid (umol/L)	318.8 ± 95.9	4.5–616
HbA1c (%)	6.0 ± 0.8	4.8–11.4
Blood glucose (mmol/L)		
0 h	5.1 ± 0.9	3.54–9.74
1 h	9.9 ± 2.9	3.03–21.18
2 h	8.7 ± 3.5	3.53–24.9
3 h	6.2 ± 3.0	2.03–22.48
Insulin (uIU/ml)		
0 h	11.4 ± 19.6	1.95–368.9
1 h	94.9 ± 77.0	9.33–755.5
2 h	102.1 ± 90.1	8.46–1,000
3 h	48.8 ± 64.3	3.41–1,000
C-peptide (ng/ml)		
0 h	2.6 ± 1.0	0.83–7.46
1 h	9.9 ± 3.9	1.61–29.75
2 h	11.7 ± 4.6	2.49–30.29
3 h	8.3 ± 3.6	2.02–21.52
HOMA-β	194.6 ± 530.6	9.98–7960
HOMA-IR	2.6 ± 4.2	0.39–76.57
ISI	77.2 ± 28.8	15.59–195.93
CSI	195.5 ± 50.7	99.98–359.44
AUC of insulin	227.0 ± 186.1	22.60–2439.95
AUC of C-peptide	27.1 ± 9.3	6.03–60.34

SD, standard deviation; Tg, thyroglobulin; Tg-Ab, antithyroglobulin antibody; TSH, thyroid stimulating hormone; TG, triglycerides; TC, total cholesterol; LDL-c, low-density lipoprotein cholesterol; HDL-c, high-density lipoprotein cholesterol; HbA1c, glycosylated hemoglobin; HOMA-β, homeostasis model assessment of β cell; HOMA-IR, homeostasis model assessment of insulin resistance; ISI, insulin sensitivity index; CSI, C-peptide sensitivity index; AUC, area under curve.

### Glucose Metabolism Index and Aggressiveness of Differentiated Thyroid Carcinoma

**Figure 1:**
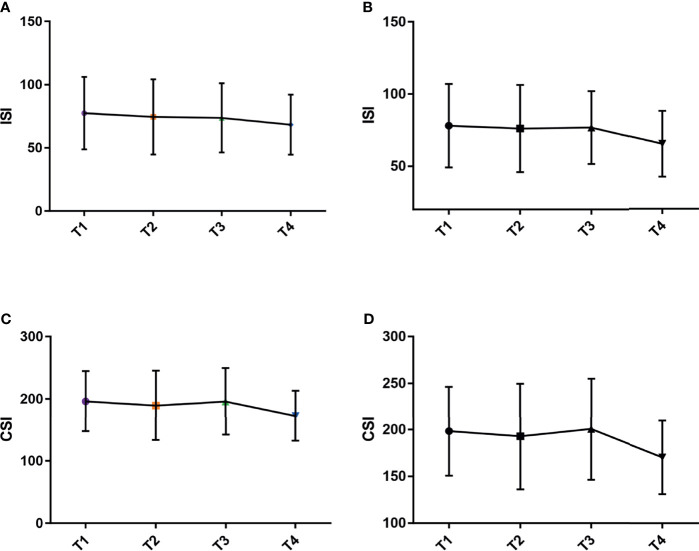
Correlation between ISI, CSI and T staging. **(A)** ISI in all patients. **(B)** ISI in patients without DM. **(C)** CSI in all patients. **(D)** CSI in patients without DM.

Spearman’s correlation analysis evaluating the association between glucose metabolism index and aggressiveness of DTC in all patients is shown in **Table 4**. Level of 2 h serum insulin (*P* = 0.041), 3 h serum insulin (*P* = 0.010), 2 h serum C-peptide (*P* = 0.042), 3 h serum C-peptide (*P* = 0.003), ISI (*P* = 0.012), CSI (*P* = 0.016), AUC of insulin (*P* = 0.031) and C-peptide (*P* = 0.029) were significantly associated with TNM staging-T. Except for the negative correlation between ISI and T staging (r = −0.170), CSI and T staging (r = −0.163), other parameters were positively correlated. Generally, the T stage increased gradually with the increment of AUC of insulin and AUC of C-peptide. ISI, an index proposed in 1999 by Matsuda and DeFronzo, was alleged to be highly correlated with the insulin sensitivity measured by the positive glucose clamp technique, and it was thought superior to HOMA-IR. It was found that the lower the insulin sensitivity, the higher the T stage, and the results showed a downward trend (**Figure 1A**). Given that the ISI may be affected by exogenous insulin, we designed CSI, the C-peptide sensitivity index, and still found the same trend as ISI (**Figure 1C**). In total, the larger the AUC of serum insulin and C-peptide, the lower the insulin sensitivity, the higher the T stage. This suggested a correlation between T stage and hyperinsulinemia and insulin resistance.

**Table 4:** Spearman’s correlation analysis evaluating the association between glucose metabolism index and aggressiveness of DTC in all patients (TNM staging-T, number of metastatic lymph nodes and capsule invasion).

	TNM staging-T		Number of metastatic lymph nodes	
	r	*P*	r	*P*
Blood glucose				
0 h	−0.051	0.451	0.079	0.226
1 h	0.107	0.115	−0.106	0.103
2 h	0.105	0.121	0.045	0.489
3 h	0.106	0.117	0.152	0.019
Insulin				
0 h	0.097	0.152	0.034	0.600
1 h	0.110	0.106	−0.157	0.015
2 h	0.138	0.041	−0.107	0.101
3 h	0.175	0.010	−0.000	0.999
C-peptide				
0 h	0.069	0.312	0.124	0.056
1 h	0.082	0.229	−0.211	0.001
2 h	0.137	0.042	−0.176	0.007
3 h	0.198	0.003	−0.049	0.450
HOMA-β	0.095	0.161	0.042	0.521
HOMA-IR	0.091	0.178	0.037	0.572
ISI	−0.170	0.012	0.073	0.261
CSI	−0.163	0.016	0.058	0.376
AUC of insulin	0.146	0.031	−0.111	0.087
AUC of C-peptide	0.148	0.029	−0.184	0.004
Delayed peak of insulin secretion	0.093	0.169	−0.014	0.843

DTC, differentiated thyroid carcinoma; HOMA-β, homeostasis model assessment of β cell; HOMA-IR, homeostasis model assessment of insulin resistance; ISI, insulin sensitivity index; CSI, C-peptide sensitivity index; AUC, area under curve. Data for Spearman’s correlation analysis was adjusted for sex, age, body weight, height, BMI, SBP, DBP, history of DM, duration of DM, smoking, drinking, HbA1c, TG, TC, LDL-c, HDL-c, uric acid, TSH, Tg, TgAb.

In addition, a delayed peak of insulin secretion was defined when the peak of insulin secretion was later than 1 h. The former was found to be positive related with capsule invasion (r = 0.206, *P* = 0.004).

Level of 3 h blood glucose (*P* = 0.019), 1 h serum insulin (*P* = 0.015), 1 h serum C-peptide (*P* = 0.001), 2 h serum C-peptide (*P* = 0.007) and AUC of C-peptide (*P* = 0.004) were statistical related with the number of metastatic lymph nodes. There was a positive correlation between the level of 3 h blood glucose and the number of metastatic lymph nodes (r = 0.152), while other indicators were negatively correlated. However, subgroup analysis to explore the correlation between the number of metastatic lymph nodes and glucose metabolism indicators (namely, ISI, CSI, AUC of insulin and C-peptide) did not find a significant linear trend.

Further subgroup analysis, based on the history of DM, found that fasting insulin (r = 0.169, *P* = 0.012), fasting C-peptide (r = 0.133, *P* = 0.049) and HOMA-IR (r = 0.162, *P* = 0.017) were all significantly positive related with T staging in patients without DM history. Negative associations were also found between ISI (r = −0.164, *P* = 0.019), CSI (r = −0.163, *P* = 0.020) and T staging. And the trend in patients without DM history was consistent with that in all patients (**Figures 1B, D**). Level of 3 h blood glucose (*P* = 0.159), 3 h serum insulin (*P* = 0.021), 3 h serum C-peptide (*P* = 0.012) and AUC of C-peptide (*P* = 0.040) were statistical related with the number of metastatic lymph nodes. In summary, in patients without a DM history, the higher insulin resistance, the lower the insulin sensitivity, the higher the T stage. At the same time, the larger the AUC of serum C-peptide, especially, glucose metabolism parameter at 3-hour after a meal, the larger number of metastatic lymph nodes.

## Discussion

DTC, especially PTC, is thought to be a tumor with a better prognosis than other types of cancer. Multifocality and metastasis have been considered as aggressive factors for PTC and are associated with the poor outcome, and even increase the risk of recurrence (15–18). Thus, it is important to explore the risk factors of multifocality, metastasis and other pathological features that may cause a poor prognosis of the disease. In our cross-section study, the known DM was about 10.1%, and this was consistent with the current prevalence of type 2 diabetes in China (11.2%) (19). However, the proportion of postprandial 2 h blood glucose ≥11.1mmol/L and HbA1c ≥6.5% was significantly higher than the known prevalence of DM. That means the prevalence of DM is considerably higher in the thyroid cancer group than that in the general population. Additionally, 87.3% of the DTC patients in this cross-section study had varying degrees of insulin resistance. Further analysis by Spearman’s correlation analysis found that higher T staging was associated with higher levels of AUC of C-peptide, and abnormal insulin/C-peptide sensitivity. A delayed peak of insulin secretion was found to be positive related with capsule invasion. Also, in patients without a DM history, insulin resistance and lower insulin/C-peptide sensitivity were statistic associated with T staging. Also, the glucose metabolism parameter at 3 h after a meal was related to a larger number of metastatic lymph nodes. In general, abnormal glucose metabolism, namely, DM, hyperinsulinemia and insulin resistance, were significantly associated with the carcinogenesis and aggressiveness of DTC.

DM and cancer are two major global health issues. Epidemiological studies found that DM, especially type 2 DM (T2DM), always accompanied with a malignant tumor (e.g., colorectal cancer (20, 21), liver cancer (22), breast cancer (23), etc.). The conflicting cumulative data indicate that DM or prediabetes may be risk factor for thyroid cancer (9, 24, 25). In addition, the duration of diabetes is presumed to be positively associated with the risk of thyroid cancer (26). Moreover, patients with T2DM and DTC had an advanced TNM stage at the time of diagnosis and increased disease-specific mortality (10). Long-term hyperglycemia, serve as a direct and only energy source for tumor cells, provide conditions for the growth of tumor cells. Shih et al. (27) reviewed the literature and proposed mechanisms linking the association between DM and thyroid cancer. DM may affect mitogenic pathway of the follicular cells through the following mechanisms. Hyperglycemia and hypertriglycemia increase oxidative stress, increased BMI will increase adipokines, antidiabetic medicines (such as sulfonylurea and insulin), contribute to the elevated insulin level, and subsequently stimulate mitogenic pathways. Increased insulin can stimulate follicular epithelial cell proliferation due to its structural similarity to insulin-like growth factor (IGF). Increased TSH acts directly on mitogenic pathways. All of the above explained the potential mechanisms from the pathophysiological perspective.

Insulin, a principal anabolic hormone, is responsible for the proper storage of nutrients after a meal. Increased levels of insulin are observed in patients with T2DM, which may be due to an increase of endogenous insulin (associated with insulin resistance) or exogenous insulin action (drugs). Previous clinical study has found that women with T2DM treated with long-term subcutaneous insulin injection have an increased incidence of breast cancer (28). Sheng et al. found that both human insulin and glargine did stimulate thyroid cell proliferation at high doses (29). From the evidence mentioned above, it is reasonable to speculate that hyperinsulinemia (both endogenous and exogenous insulin) may play a role in cancer. As known, an increment of insulin and the IGF-1 are related to cancers, such as breast cancer and colon cancer (30, 31). For the structural homology and affinity with IGF-1 receptor, insulin may also play an important role in cell proliferation and apoptosis. When culturing follicular cells, TSH combined with insulin resulted in a significant increment of cell numbers compared to TSH alone, suggesting a mimic action of the insulin to IGF-1 (regulate the growth of follicular cells) (32, 33). Therefore, insulin may play a vital role in the carcinogenesis of the thyroid.

Insulin resistance, a typical feature of patients with T2DM, is considered to be a risk factor for a variety of cancers, such as hepatocellular, breast cancer (34, 35). Although several studies have found the relationship between insulin resistance and thyroid nodules or thyroid cancer (11, 36), the role of insulin resistance in carcinogenesis of thyroid is still debatable, some studies have found a correlation (11–13, 37, 38), in contrast, others did not find positive results (15, 39–41). In our study, ISI was associated with the aggressiveness of thyroid cancer, but HOMA-IR was not. So, the different indicators of insulin resistance/insulin sensitivity used in different studies may be the reason for the discrepancy in previous studies. To date, rare clinical studies concluded the link between insulin resistance and thyroid cancer through direct exposure.

Recently, Elbasan et al. (42) investigated the association of T2DM on the histological aggressiveness in DTC patients, and did not found the additive effect of T2DM on DTC aggressiveness. Guo et al. (43) conducted a study in China and found that hyperinsulinemia might be the risk factor of PTC, but not disease severity. On the contrary, HOMA-IR index was reported to affect the tumor diameter of PTC (38). A clinical study conducted in Korean women found that increased serum glucose, insulin levels, and a higher HOMA-IR were associated with the multifocality of PTC (12), which suggested the correlation of hyperinsulinemia and/or insulin resistance and the aggressiveness of PTC. In our cross-section study, we found the insulin resistance (lower ISI and CSI) and a delayed peak of insulin secretion were significantly associated with the aggressiveness of DTC. In addition, patients without a DM history, HOMA-IR, ISI and CSI were statistic associated with T staging, and the glucose metabolism parameter at 3 h after a meal was related to a larger number of metastatic lymph nodes. But there still need further clinical studies with a larger sample size to clarify the relationship and fundamental researches to elucidate the mechanisms.

## Conclusion

In conclusion, abnormal glucose metabolism, namely, DM, hyperinsulinemia and insulin resistance, were significantly associated with the carcinogensis and aggressiveness of DTC. Moreover, further prospective clinical trials and basic research are needed to confirm the results.

## Data Availability Statement

The raw data supporting the conclusions of this article will be made available by the authors, without undue reservation.

## Ethics Statement

The studies involving human participants were reviewed and approved by The Ethics Committee of the First Affiliated Hospital of Shandong First Medical University & Shandong Provincial Qianfoshan Hospital. The patients/participants provided their written informed consent to participate in this study.

## Author Contributions

JZ, LL and JD conceived and designed the study. JZ, YT, ZJ, and JY collected and analyzed the data. JZ wrote the paper. LL and JD supervised the whole study and revised the manuscript. All authors listed have made a substantial, direct, and intellectual contribution to the work and approved it for publication.

## Funding

This work was supported by the Shandong Provincial Natural Science Foundation of China Grants [grant number ZR2019PH025]. They supported the study design, the collection, analysis and interpretation of data, the writing of the report, and the decision to submit the article for publication.

## Conflict of Interest

The authors declare that the research was conducted in the absence of any commercial or financial relationships that could be construed as a potential conflict of interest.

## Publisher’s Note

All claims expressed in this article are solely those of the authors and do not necessarily represent those of their affiliated organizations, or those of the publisher, the editors and the reviewers. Any product that may be evaluated in this article, or claim that may be made by its manufacturer, is not guaranteed or endorsed by the publisher.
